# Mathematical modeling of GATA-switching for regulating the differentiation of hematopoietic stem cell

**DOI:** 10.1186/1752-0509-8-S1-S8

**Published:** 2014-01-24

**Authors:** Tianhai Tian, Kate Smith-Miles

**Affiliations:** 1 School of Mathematical Sciences, Monash University, Melbourne, VIC 3800, Australia

## Abstract

**Background:**

Hematopoiesis is a highly orchestrated developmental process that comprises various developmental stages of the hematopoietic stem cells (HSCs). During development, the decision to leave the self-renewing state and selection of a differentiation pathway is regulated by a number of transcription factors. Among them, genes *GATA-1 *and *PU.1 *form a core negative feedback module to regulate the genetic switching between the cell fate choices of HSCs. Although extensive experimental studies have revealed the mechanisms to regulate the expression of these two genes, it is still unclear how this simple module regulates the genetic switching.

**Methods:**

In this work we proposed a mathematical model to study the mechanisms of the GATA-PU.1 gene network in the determination of HSC differentiation pathways. We incorporated the mechanisms of GATA switch into the module, and developed a mathematical model that comprises three genes *GATA-1, GATA-2 *and *PU.1*. In addition, a novel multiple-objective optimization method was designed to infer unknown parameters in the proposed model by realizing different experimental observations. A stochastic model was also designed to describe the critical function of noise, due to the small copy numbers of molecular species, in determining the differentiation pathways.

**Results:**

The proposed deterministic model has successfully realized three stable steady states representing the priming and different progenitor cells as well as genetic switching between the genetic states under various experimental conditions. Using different values of GATA-1 synthesis rate for the GATA-1 protein availability in the chromatin sites during the time period of GATA switch, stochastic simulations for the first time have realized different proportions of cells leading to different developmental pathways under various experimental conditions.

**Conclusions:**

Mathematical models provide testable predictions regarding the mechanisms and conditions for realizing different differentiation pathways of hematopoietic stem cells. This work represents the first attempt at using a discrete stochastic model to realize the decision of HSC differentiation pathways showing a multimodal distribution.

## Background

Hematopoiesis is a highly orchestrated developmental process that comprises the proliferation, differentiation and maturation of a very small population of self-renewing, pluripotent hematopoietic stem cells (HSC) for producing different types of blood cells, including erythrocyte, megakaryocyte, granulocyte, and macrophage [1,2]. During development, the decision to leave the self-renewing state and selection of a differentiation pathway are regulated by transcription factors (TFs) [3-6]. Intense experimental studies during the past two decades have suggested that tight regulation of HSC differentiation is controlled by the interaction of a number of genetic and epigenetic regulators of gene transcription, including the two TFs PU.1 and GATA-1. Although the precise mechanisms to initiate the transcriptional cascade leading to different differentiated cells are not clear currently, experimental studies have established that both *PU.1 *and *GATA-1 *'autoregulate' themselves, i.e. they stimulate their own production, as well as they are mutually antagonistic, i.e. they repress the production of each other [7-9]. In the erythrocyte/megakaryocite lineage high expression levels of gene *GATA-1 *and low levels of *PU.1 *were detected [6,10]; conversely, in the granulocyte/macrophage lineage higher expression levels of *PU.1 *and low levels of *GATA-1 *were measured [5,11]. However, the initial progenitor cells stay in the third state that has low-level activation of both genes *PU.1 *and *GATA-1*. When a progenitor cell differentiates, it transitions from the initial indeterminate state into one of two differentiated states with high expression levels of either gene *GATA-1 *or *PU.1*. Thus the full understanding of the PU.1 and GATA-1 interaction is important for studying of the differentiation process of HSCs, which may be very useful in the clinical application of "differentiation therapy" for re-establishing the correct expression of *PU.1 *and/or *GATA-1 *within immature leukemic cells [12].

To accurately describe the regulatory mechanisms controlling HSC differentiation, the important aspects that any mathematical model of the GATA-1-PU.1 network must include are an indeterminate state for the progenitor cells and two stable attractors of the dynamical system for the differentiated lineages. Since the first modeling attempt to study the regulation in the GATA-1-PU.1 network [13], a number of mathematical formalisms have been developed to realize the three stable steady states in HSCs. For example, Huang et al. used the Hill function with high co-operativity coefficients to qualitatively compare computer predictions with experimental evidence [14], giving support to the idea that lineage choice occurs as a two stage process, first priming and then differentiating. To remove the requirement of high co-operativity, Chickarmane et al. assumed that the autoregulation at both PU.1 and GATA-1 occurs through the binding of monomers [15]. It was predicted that an additional mechanism should be involved in the repressive interaction to create a bistable switch, and therefore a third unspecified gene was introduced to create bistability. Alternatively, a mathematical model was proposed to include the dynamics of the inactive heterodimer GATA-1-PU.1 and the Michaelis-Menten function was used to represent the low co-operativity [16]. We have designed a model by separating the strength of co-operativity for autoregulation and repression, and successfully realized a rich variety of systems behavior that have been found cross the existing models [17]. Recently Huang and collaborators proposed a general model that assumed no explicit interaction between the two genes; and realized a degenerated steady state via a new type of bifurcation [18]. Since the assumptions in these models are based on either unrealistic high co-operativity or unspecified gene, additional mechanisms are needed to accurately describe the differentiation decision of HSCs.

GATA-2 is one of the six members of the hematopoietic GATA factor family and is most abundantly expressed in HSCs as well as in immature progenitors in hematopoietic lineages [10]. Previous studies demonstrated a critical role of *GATA-2 *in the emergence and maintenance of HSCs [19]. In addition, strict regulation of genes *GATA-1 *and *GATA-2 *is critical for proper lineage commitment and development of erythroid cells. It was reported that GATA-2 directly activated *GATA-1 *expression in early erythroid progenitors, and then GATA-1 accelerated its expression after its own expression has been initiated [20]. The gene expression process involving GATA-1-mediated displacement of GATA-2 from chromatin is termed a GATA switch [21-23]. This "GATA factor switch" suggests a model whereby GATA-2 and GATA-1 sequentially bind the same *cis-*elements with activating and repressive effects, respectively. During GATA factor switch, the *GATA-1 *expression will increase due to the reciprocal decrease of GATA-2, which leads progenitor cells to adopt an erythroid lineage. However, when GATA-1 is absent or its expression is delayed, the reduction of GATA-2 will increase the expression levels of *PU.1 *and leads progenitor cells to adopt a myeloid lineage [24,25]. Therefore the dynamic expression patterns of *GATA-1 *and *GATA-2 *may influence the erythroid-myeloid cell-fate selection by regulating the expression of gene *PU.1 *[22]. Although accumulating experimental evidence has suggested the important role of gene *GATA-2 *in regulating the cell-fate selection, only a simple Boolean network model has been proposed recently to include the regulatory function of gene *GATA-2 *[26]. The kinetic dynamics of GATA-2 and in particular the function of GATA switch has not been systematically studied so far.

Recent experimental studies have demonstrated that gene expression is a stochastic process. Key species of molecules such as DNA and mRNA may have small copy numbers, and the change of their copy numbers may cause significant variations of the system dynamics [27-30]. In particular, it has been shown that a variety of lineage-restricted genes in HSCs were expressed at low levels [31]. A recent study directly demonstrated that stochastic oscillation expression of lineage-associated genes could drive cell-fate commitment [32]. Accumulating experimental evidence also suggested that stem cells are heterogeneous, with cells moving between two or more metastable states [33]. Recently a minimal model has been designed by combining cell-extrinsic and cell-intrinsic elements of regulation to understand how both instructive and stochastic events could inform cell commitment decision in hematopoiesis [34]. However, compared with the advances in developing various stochastic models to investigate the key functions of noise in genetic and cellular processes [35-37], the critical role of noise in determining the stem cell differentiation has not been well established. This work is aimed at developing the first stochastic model to explore the critical function of GATA switch and noise in determining the differentiation pathways of HSCs.

## Methods

### Mathematical model of GATA-PU.1 gene network

**Figure 1:**
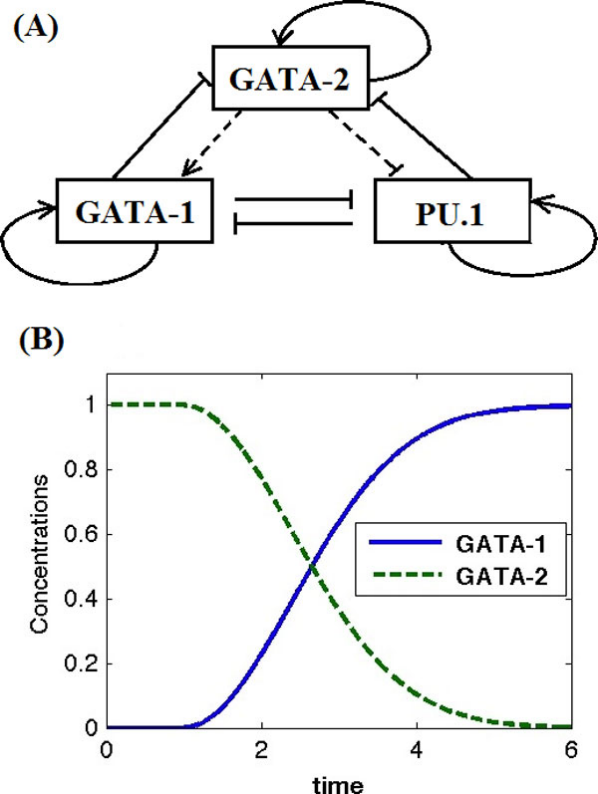
**Diagram of the GATA-PU.1 regulatory network**. (A) The genetic regulation of the GATA-PU.1 network. (B) (Normalized) expression levels of genes *GATA-1 *and *GATA-2 *during GATA switch. Time was in arbitrary unit.

In this work we propose a mathematical model for the GATA-PU.1 regulatory network including genes *GATA-1, GATA-2 *and *PU.1 *(Figure 1A). It was assumed that each of these three genes activates their own expression through positive autoregulation. In addition, TF GATA-2 activates the expression of gene *GATA-1 *but inhibits the expression of gene *PU.1*, but the function of GATA-2 to regulate the expressions of genes *GATA-1 *and *PU.1 *is moderate [24]. Thus the expression levels of three genes *GATA-1, GATA-2 *and *PU.1 *may maintain at an intermediate state, which is compatible to the steady state of lineage priming. In addition, GATA-1 inhibits the expression of *GATA-2 *and *PU.1*, whereas PU.1 inhibits the expression of *GATA-1 *and *GATA-2*. Detailed assumptions of regulatory mechanisms and the list of chemical reactions are given in Additional file 1.

Based on the GATA switch model (Figure 1B), GATA-2 localizes at the chromatin sites in early stage erythroblasts. When the expression levels of GATA-1 increase as erythropoiesis progresses, GATA-1 displaces GATA-2 from chromatin sites and often (but not always) instigates a distant transcriptional output [21]. Although remaining in the cell, TF GATA-2 in fact is unable to access the chromatin sites. To model this spatial regulatory mechanism, it was assumed that GATA-2 proteins degrade during the process of GATA switch, which was realized by a large degradation rate constant $k_{2}^{*}$ of GATA-2 in Eq. (1). Simultaneously, the concentration of GATA-1 increases by using an additional synthesis rate of GATA-1, which is proportional to the removal of GATA-2.

Experiments also showed that, when GATA-1 was not present, the shRNA knockdown of GATA-2 increases the expression of gene *PU.1 *and reprograms the cells to become macrophages [24]. To realize genetic switching, the mechanism of the GATA switch and that of PU.1 knockdown were included in a single framework. Thus the knockdown of GATA-2 was realized by a large degradation rate constant $k_{2}^{*}$ of GATA-2 during a particular time period. In addition, a very small synthesis rate of GATA-1 during that time period means the absence of GATA-1 protein in the DNA promoter region. Furthermore, for simplicity, the basal expression rates of these three genes are assumed to be zero. We use the Shea-Ackers formalism, which is a widely used thermodynamic approach, to represent the gene expression based on the structure of transcription machinery [38]. All these assumptions led to the following model to realize the genetic switching of the GATA-PU.1 regulatory network, given by

(1) $$\begin{array}{c} \frac{dx}{dt}=\frac{a_{1}x+a_{2}y}{a_{3}+a_{4}x+a_{5}y+a_{6}z+a_{7}xz}-k_{1}x+\mu k_{2}^{*}y\\ \frac{dy}{dt}=\frac{b_{1}y}{b_{2}+b_{3}x+b_{4}y+b_{5}z+b_{6}yz}-k_{2}y-k_{2}^{*}y\\ \frac{dc}{dt}=\frac{c_{1}z}{c_{2}+c_{3}x+c_{4}y+c_{5}z+c_{6}xz+c_{7}yz}-k_{3}z \end{array}$$

where *x, y *and *z *are the concentrations of TFs GATA-1, GATA-2 and PU.1, respectively, *a*_1_, *b*_1 _and *c*_1 _represent the expression rates of genes *GATA-1, GATA-2 *and *PU.1 *auto-regulated by itself, respectively, *a*_2 _is the expression rate of gene *GATA-1 *regulated by TF GATA-2, *k*_1_, *k*_2 _and *k*_3 _are the degradation rates of TFs GATA-1, GATA-2 and PU.1, respectively, $k_{2}^{*}$ is the degradation rate constant to represent the displacement of GATA-2 proteins,

(2) $$k_{2}^{*}=\left\{\begin{array}{cc} k_{20} & t\in \left[t_{1},t_{2}\right]\\ 0 & else \end{array}\right)$$

during the time period [*t*_1_, *t*_2_] , and *μ *is a parameter to adjust the availability of GATA-1 proteins in the chromatin sites. The GATA switch model is realized by using $k_{2}^{*}>0$, *μ *> 0 with a moderate value of parameter *μ *(e.g. *μ *= 1). In contrast, the knockdown of GATA-2 is realized by using $k_{2}^{*}>0$, *μ *= 0. Another realization of the GATA-2 knockdown is to use rate constants $k_{2}^{*}$ = 0, *b*_1 _= 0 for the expression of gene *GATA-2*, namely to block the synthesis process of GATA-2 but maintain the degradation process of GATA-2 unchanged. Numerical results did not show any substantial difference between the computer simulations of these two types of realization (results not shown). Finally we note that the proposed model (Eq. 1) includes a recently designed model for the GATA-1-PU.1 module [18] as a special case if gene *GATA-2 *is removed from the system.

### Model parameters estimated from experimental data

There are 23 rate constants in the proposed mathematical model (Eq. 1). Part of the parameters was determined by the following experimental data.

(1) The half-life of GATA-1 protein is one hour [39]. In addition, the half-life of GATA-2 is 30 min [40] that was confirmed by another observation in [33] therein. The half-life of PU.1 in Mel cell is ~2.4 h [41]. Thus the protein degradation rates constants are *λ*_1 _= 0.6931/*h*, *λ*_2 _= 1.3863/*h*, *λ*_3 _= 0.2888/*h*.

(2) The disassociate rate of GATA-1 binding to its DNA promoter is *K_d _*= 2.8 nM, which is more stable than the binding of GATA-2 to its promoter site *K_d _*= 4.4 nM [42]. In addition, the disassociate rate of PU.1 binding to its DNA promoter is *K_d _*= 170 nM [43]. These rates were used to determine coefficients *a*_4_, *b*_4_, and *c*_5_.

(3) In addition, the heterodimer GATA-1-PU.1 has a 3-fold increase of the binding rate constant over GATA-1 to DNA [7]. It was assumed that *a*_7 _= 3*a*_4_.

(4) The disassociation rates of GATA-1 and GATA-2 binding to the DNA promoter sites are very close to each other [44]. It is assumed that the binding rates of GATA-1 and GATA-2 to the same DNA binding sites are the same, namely *b*_6 _= *a*_7_, and *c*_7 _= *c*_6_.

### Multiple-objective optimization approach

**Figure 2:**
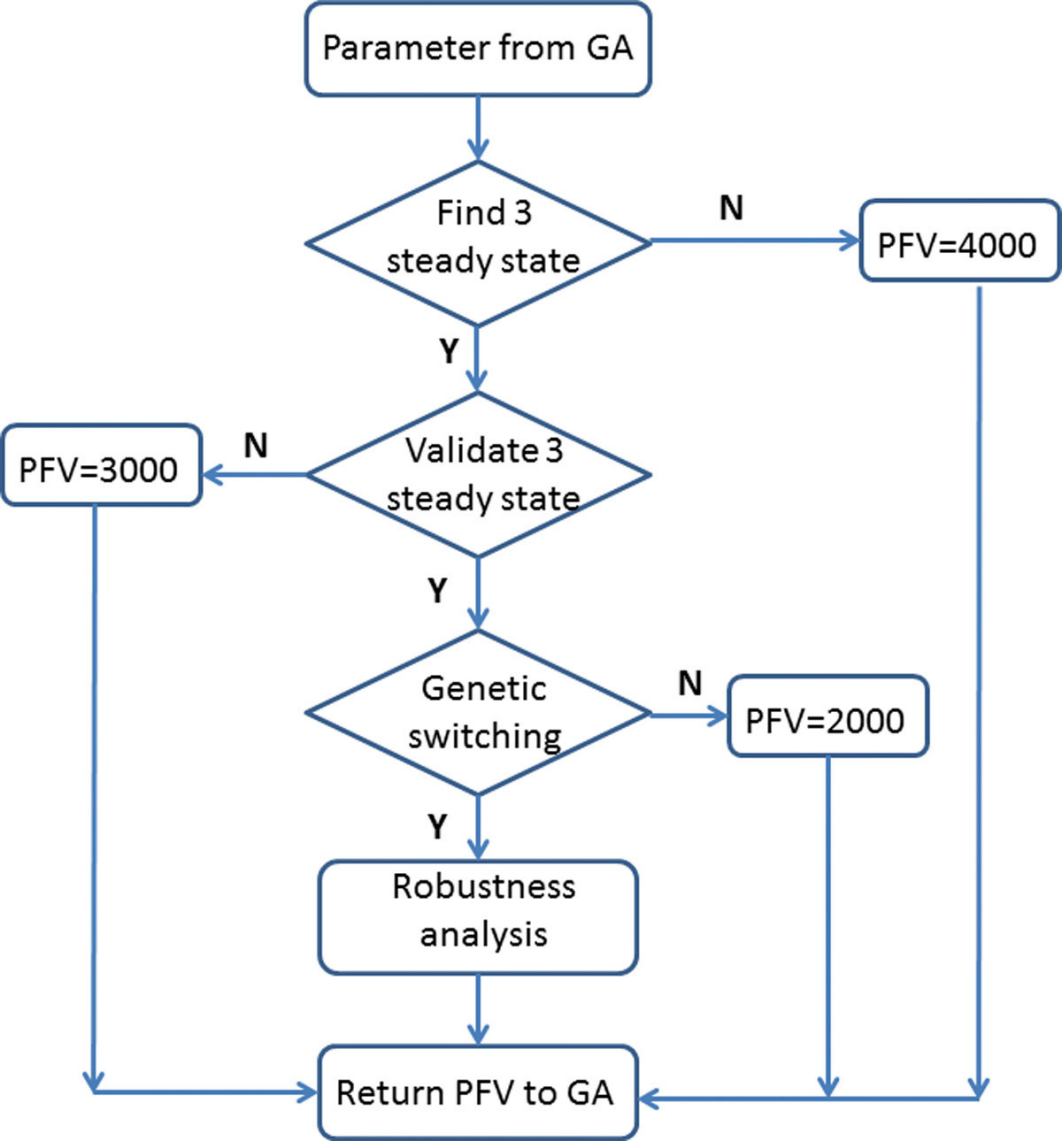
**Schematic representation of the multiple-objective optimization algorithm for estimating model parameters**. This algorithm includes five major steps, namely to generate initial sets of model parameters from a genetic algorithm (GA) in Step 1; to find the three steady states of the model with a particular set of parameters in Step 2; to validate the existence of the three steady states in Step 3; to examine the existence of genetic switching in Step 4; and to conduct robustness analysis of the model based on the property of genetic switching in Step 5. Finally the penalty function value (PFV) is sent back to the GA for the selection of the optimal set of parameters.

To infer the unknown parameters in model (Eq. 1), we designed a novel multiple-objective optimization approach to estimate unknown model parameters (Figure 2). The criteria in this approach include the conditions of tristability, genetic switching and robustness property of the model. This approach includes the following major steps:

**Step 1. Generate a set of model parameters in the GA**. We first used the uniform distributed random variable *U*(0,1) generated in the genetic algorithm (GA) to determine the initial model rate constants. Note that the mutation manipulation in the GA was carried out on the random samples rather than model parameters. Since there are 7 restricted conditions for the existence of stable steady states (Eq. 6~8), other parameters were directly determined by the random samples *a_i _*= *r_i_*, where *r_i _*~ *U*(0,1). Seven parameters were determined by the random samples together with the restricted conditions. For example, according to condition (Eq. 7), parameter *b*_3 _is determined by

$$b_{3}=\frac{1}{r_{10}x_{1}^{*}}\left[\frac{b_{1}}{k_{2}}-b_{2}\right]$$

where *r*_10 _~ *U *(0,1) and $x_{1}^{*}$ is the steady state (Eq. 4). In addition, based on condition (Eq. 6), the four synthesis rates were also determined by a factor *k*,

$$a_{1}=\frac{k_{1}a_{3}}{r_{1}}\times k,\;\;\;\;a_{2}=r_{2}k,\;\;\;\;\;\;b_{1}=\frac{k_{2}b_{2}}{r_{9}}\times k,\;\;\;\;\;c_{1}=\frac{k_{3}c_{2}}{r_{16}}\times k,$$

where *r_i _*~ *U*(0,1). We tested different values of *k *in order to realize genetic switching. When *k *is not large (1 < k < 100), we failed to find a set of parameters that could realize genetic switching. Thus in this work we used k = 1000 to search the unknown synthesis rate.

**Step 2. Find the third steady state**. The generated parameter set in Step 1 ensures the existence of the two stable steady states in which either GATA-1 or PU.1 has high expression levels. To find the third stable steady state representing the primed progenitor state, we used MATLAB function *fsolve.m *to solving the nonlinear system for the steady state of the model (Eq. 1). If we can find the third stable steady state, we go to Step 3. Otherwise, we set the penalty function value to 4 and go to Step 6.

**Step 3. Validate the existence of three steady states**. Since the third steady state found in Step 2 was obtained by a numerical method, it may not exist due to the computational error. To examine the existence of the three steady states, we perturbed each steady state using samples of the uniformly distributed random variable and used the perturbed steady state as the initial condition to simulate system (Eq. 1). If the simulation converges to the steady state, it means the steady state exists, and then we go to Step 4. Otherwise, we set the penalty function value to 3 and go to Step 6.

**Step 4. Validate the existence of genetic switching**. We next examined whether the system can realize genetic switching using the mechanisms of GATA switch and GATA-2 knockdown. The large degradation rate constant of GATA-2 in model (Eq. 1) for these two mechanisms is

$$k_{2}^{*}=\left\{\begin{array}{c} 20,\;\;\;\;500\leq t\leq 1500\\ \;0,\;\;\;\;\;\;\;\;\;\;\;\;\;\;\text{else}\text{.} \end{array}\right)$$

Simultaneously, the additional synthesis rate constant of GATA-1 in model (Eq. 1) is

$$\mu =\left\{\begin{array}{c} 1,\;\;\;\;\;\;\;\;\;\;\;\text{GATA}\;\text{switch}\\ 0,\;\;\;\;\;\text{GATA - 2}\;\text{knockdown}. \end{array}\right)$$

If the system realizes genetic switching through these two mechanisms, go to Step 5. Otherwise, set the penalty function value to 2 and go to Step 6.

**Step 5. Robustness analysis**. The inferred parameter set now satisfies the requirement for realizing three stable steady states and genetic switching. Next we used robustness property of the system as an additional criterion to choose the optimal model parameters. We tested the robustness property of mathematical model using the perturbed model parameters [45,46]. Each parameter in the model was perturbed by a uniformly distributed random number

$$a_{jk}=a_{j}\left(1\;+\;\sigma^{*}\left(U_{jk}-0.5\right)\right),\;\;\;j=\text{1},\ldots ,\text{ 23},k=\text{1},\ldots ,\text{1}000,$$

where *U_jk _*~ *U*(0,1) and *σ *= 0.5 is the perturbation strength. For each set of model parameters, we obtained 1000 sets of perturbed parameters and then examined whether the mathematical model with the perturbed model parameters still maintained the three steady states. The model with a particular set of model parameters is more stable if the model maintains the three steady states with more sets of perturbed model parameters. To make an unbiased comparison, we used the same random samples *U_jk _*for different sets of parameters. The penalty function value at this step is the percentage of the parameter sets (from the 1000 sets of perturbed parameters) with which the model does not maintain the three steady states. Thus a smaller value of penalty function means better robustness property of the model.

**Step 6. Return the value of penalty function to the genetic algorithm**.

Note that there are four penalty functions in this proposed inference method, namely the existence of three steady states in Step 2 (denoted as event *O*_1_), validation of these three steady states in Step 3 (denoted as *O*_2_), validation of the existence of genetic switching in Step 4 (denoted as *O*_3_), and robustness property of the model over 1000 perturbed sets of model parameters in Step 5 (denoted as *O*_4_). Thus, if a numerical test in Steps 2~4 is not satisfied, the penalty score of that step should be larger than the maximal score of the next step. Since the maximal penalty score of Step 5 is set to unit one if none of the perturbed parameter set maintains genetic switching, the scores of the first three penalty functions are set to 4, 3 and 2, respectively. Using the notations of objective functions [47], the multi-objective function is represented by

$$\text{min}\left\{\text{4}P\left(O_{\text{1}}\right)+\text{3}P\left(O_{\text{2}}|P\left(O_{\text{1}}\right)=0\right)+\text{2}P\left(O_{\text{3}}|P\left(O_{\text{2}}\right)=0\right)+P\left(O_{\text{4}}|P\left(O_{\text{3}}\right)=0\right)\right\}.$$

Here the probability of objective *O*_1 _is defined by, for example,

$$P\left(O_{1}\right)=\left\{\begin{array}{c} 0\;\;\;\;\text{existing three steady states}\\ \text{1 }\;\;\;\;\;\;\;\;\;\;\;\;\;\text{otherwise} \end{array}\right)\text{. }$$

Similar definitions are applied to the probabilities of objectives *O*_2 _and *O*_3_.

## Results

### Steady states of the mathematical model

When $k_{2}^{*}$ = 0, the proposed mathematical model (Eq. 1) has up to four steady states. When *k*_1_*a*_4 _≠ 0 and *k*_3_*c*_5 _≠ 0, three steady states can be obtained analytically, given by

(3) $$\text{(}x_{0},y_{0},z_{0}\text{)}=\left(0,0,0\right)$$

(4) $$\left(x_{\text{1}},y_{\text{1}},z_{\text{1}}\right)=\left(\frac{a_{\text{1}}-k_{\text{1}}a_{\text{3}}}{k_{\text{1}}a_{\text{4}}},0,0\right)$$

(5) $$\text{(}x_{2},y_{2},z_{2}\text{)}=\left(0,0,\frac{c_{\text{1}}-k_{\text{3}}c_{\text{2}}}{k_{\text{3}}c_{\text{5}}}\right).$$

The existence conditions of these three steady states are given in Theorem 1.

**Theorem 1 **(1) The trivial steady state (Eq. 3) is unstable if any one of the following conditions is satisfied

(6) $$a_{\text{1}}>a_{\text{3}}k_{\text{1}},\;b_{\text{1}}>b_{\text{2}}k_{\text{2}},\;\text{and}\;c_{\text{1}}>c_{\text{2}}k_{\text{3}}.$$

(2) The steady state with high expression levels of gene *GATA-1 *(Eq. 4) is stable if the following conditions are satisfied

(7) $$b_{\text{1}}<k_{\text{2}}\left(b_{\text{2}}+b_{\text{3}}x_{\text{1}}\right),\text{ and}\;c_{\text{1}}<k_{\text{3}}\left(c_{\text{2}}+c_{\text{3}}x_{\text{1}}\right).$$

(3) The steady state with high expression levels of gene *PU.1 *(Eq. 5) is stable if the following conditions are satisfied

(8) $$a_{\text{1}}<k_{\text{1}}\left(a_{\text{3}}+a_{\text{6}}z_{\text{2}}\right),\text{ and}\;b_{\text{1}}<k_{\text{2}}\text{(}b_{\text{2}}+b_{\text{5}}z_{\text{2}}\text{)}.$$

The proof of this theorem is provided in the Additional file 1.

However, it is difficult to derive an analytical expression of the fourth potential steady state. For a given set of model parameters, we have to examine the existence of the fourth steady state numerically on a case-by-case basis. The existence conditions of the stable steady states will be used as criteria to search the unknown model parameters.

### Inference of model parameters

The proposed model (Eq. 1) has 20 unknown parameters by setting *a*_3 _= *b*_2 _= *c*_2 _= 1. We first estimated the values of 9 model parameters, namely (*a*_4_, *a*_7_, *b*_4_, *b*_6_, *c*_5_, *k*_1_, *k*_2_, *k*_3_) and (*c*_6 _= *c*_7_) from the published experimental data discussed in the model section. Since there is not any published data for the temporal dynamics of gene expression, we used the designed multiple-objective optimization approach to estimate the remaining 11 unknown parameters (Figure 2). We used the genetic algorithm as an effective tool to search the optimal model parameters to realize genetic switching. A MATLAB toolbox developed by Chipperfield et al. [48] was used to infer the unknown model parameters. This toolbox used MATLAB functions to build a set of versatile routines for implementing a wide range of genetic algorithms. The initial estimate of rate constants can be changed by using different random seeds in the MATLAB toolbox, leading to different final estimates of the rate constants [49]. The genetic algorithm was run over 100 generations for each estimate, and we used a population of 1000 individuals in each generation. Our tests showed that a smaller population of individuals (i.e. 100 or 200) failed to produce an estimate of model parameters with which the model has tristability property. We implemented the genetic algorithm with different initial kinetic rates and obtained a number of estimated parameter sets. The parameter set having the best robustness property was selected as the final estimate.

**Figure 3:**
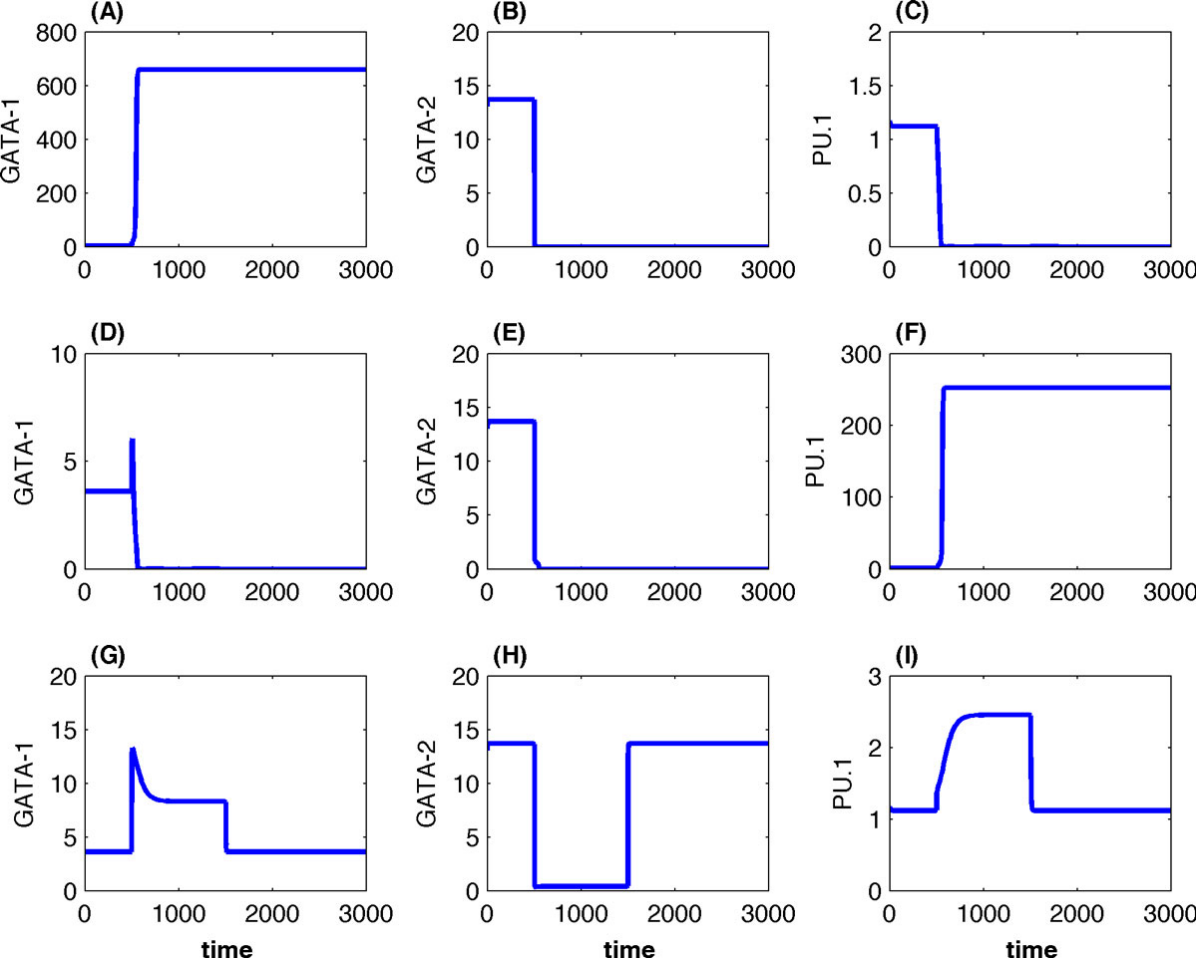
**Genetic switching of the deterministic GATA-PU.1 regulatory network**. (A, B, C) A successful switching leading to high expression level of GATA-1. The mechanism of GATA-switching was realized by a large synthesis rate (*μ *= 1) in (10). (D, E, F) A successful switching leading to high expression level of gene PU.1 if GATA-1 was knocked down by blocking the expression of GATA-1 (*μ *= 0). (G, H, I) An unsuccessful switching using an intermediate synthesis rate (*μ *= 0.32). Parameters of the model are: (*a*_1_, *a*_2_, *a*_3_, *a*_4_, *a*_5_, *a*_6_, *a*_7_) = (731.7409, 856.1247, 1, 1.6, 398.9719, 44.8982, 53.0), (*b*_1_, *b*_2 _,*b*_3_, *b*_4_, *b*_5_, *b*_6_) = (18470.6419, 1, 37.3615, 942.1939, 55.0375, 53.0), (*c*_1_, *c*_2_, *c*_3_, *c*_4_, *c*_5_, *c*_6_, *c*_7_) = (12391.1968, 1, 710.4490, 522.4385, 170.0, 1700.0, 1700.0), and (*k*_1_, *k*_2 _, *k*_3_) = (0.6931, 1.3863, 0.2888).

Figure 3 gives simulations of the deterministic model (Eq. 1) using the degradation rate constant of GATA-2 (Eq. 2) and different values of the GATA-1 synthesis rate *μ*. The concentrations of GATA-2 decreased substantially during *t *∈ [500, 1500] when a large degradation rate $k_{2}^{*}$ = 6 was applied during that time period. Figures 3A, 3B and 3C give a simulation using the GATA switch module that was realized by *μ *= 1. When the concentration of GATA-2 decreases during *t *∈ [500, 1500] in Figure 3B, GATA-1 reaches the high expression level quickly in Figure 3A. However, when GATA-1 is absent (*μ *= 0) and GATA-2 is knockdown, Figures 3D, 3E and 3F show a simulation leading to the high expression level of PU.1. The third scenario is that the expression of GATA-1 maintains at a low level during the period of GATA-2 decreases when *μ *= 0.32. In this case the expression levels of these three genes in Figures 3G, 3H and 3I maintain at an intermediate state that is dependent on the value of *μ*. Neither GATA-1 nor PU.2 reached the steady state of high expression levels. When the expression level of GATA-2 returned to the normal state at *t *= 1500, the system also returned to the primed steady state, which is an unsuccessful genetic switching.

**Figure 4:**
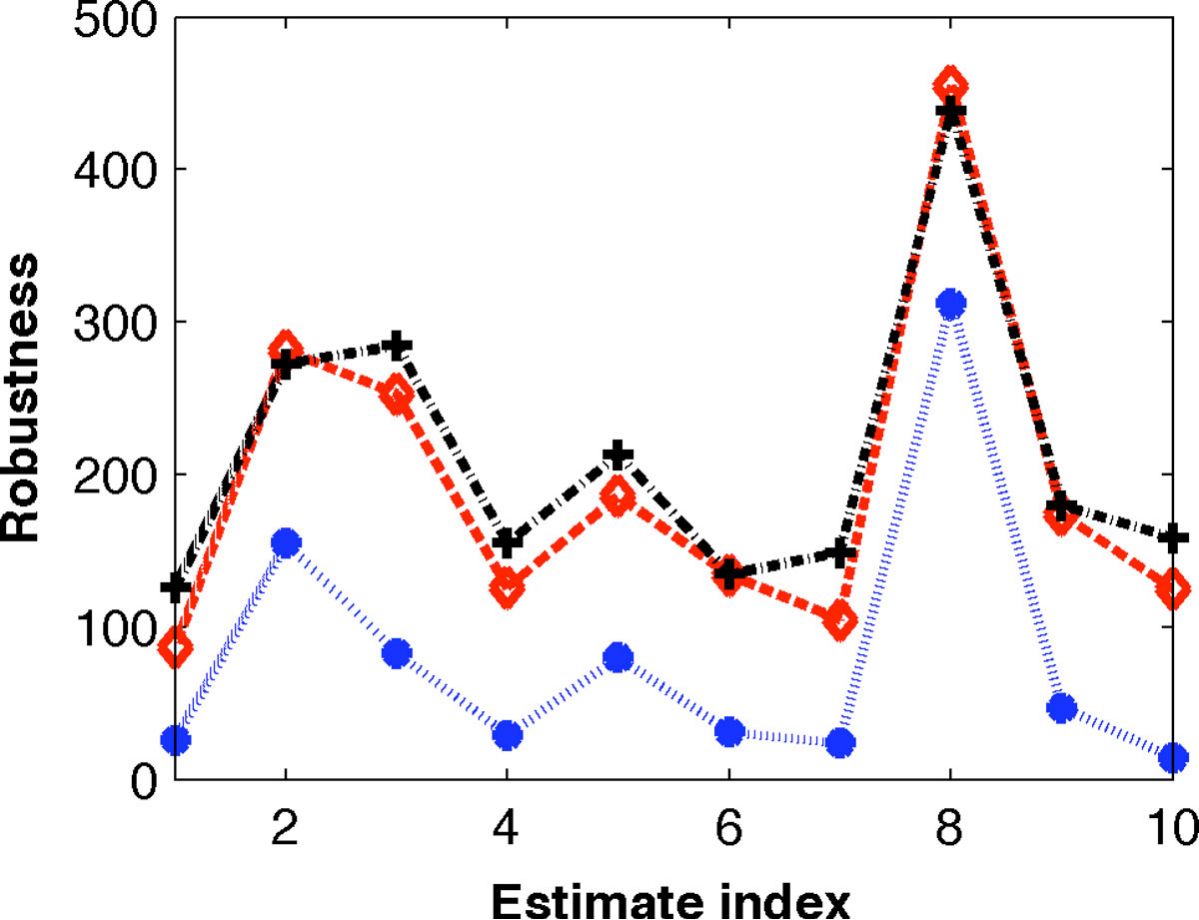
**Fractions of the perturbed model parameters that maintain the tristability property**. Test is based on the 10 sets of estimated model parameters. (Star: perturbation strength *σ *= 0.3, circle: *σ *= 0.5, plus: averaged fractions over *σ *= 0.1 ~ 1).

When inferring the model parameters, we only tested the robustness property of the model with perturbation strength *σ *= 0.5 (see Step 5 in the Optimization Approach section). The next question is whether different values of the perturbation strength may lead to different selections of the estimated model parameters. To answer this question, we selected 10 sets of the estimated model parameters that have better robustness properties than the others, and examined the robustness property of the model using different perturbation strengths ranging from 0.1 to 1. Figure 4 shows that, the robustness property of the model with a smaller strength (*σ *= 0.3) is slightly different from that using the perturbation strength (*σ *= 0.5) because only a small numbers of perturbed parameters sets did not maintain the tristability property. However, when *σ *≥ 0.4, the robustness property based on different values of perturbation strength *σ *is consistent with that using (*σ *= 0.5). Figure 4 also presents the averaged robustness properties based on 10 values of the perturbation strength (*σ *= 0.1, 0.2, ..., 1), which is well consistent with that using *σ *= 0.5. Thus it is suggested that the inferred model parameter is independent of the perturbation strength.

### Bifurcation analysis

**Figure 5:**
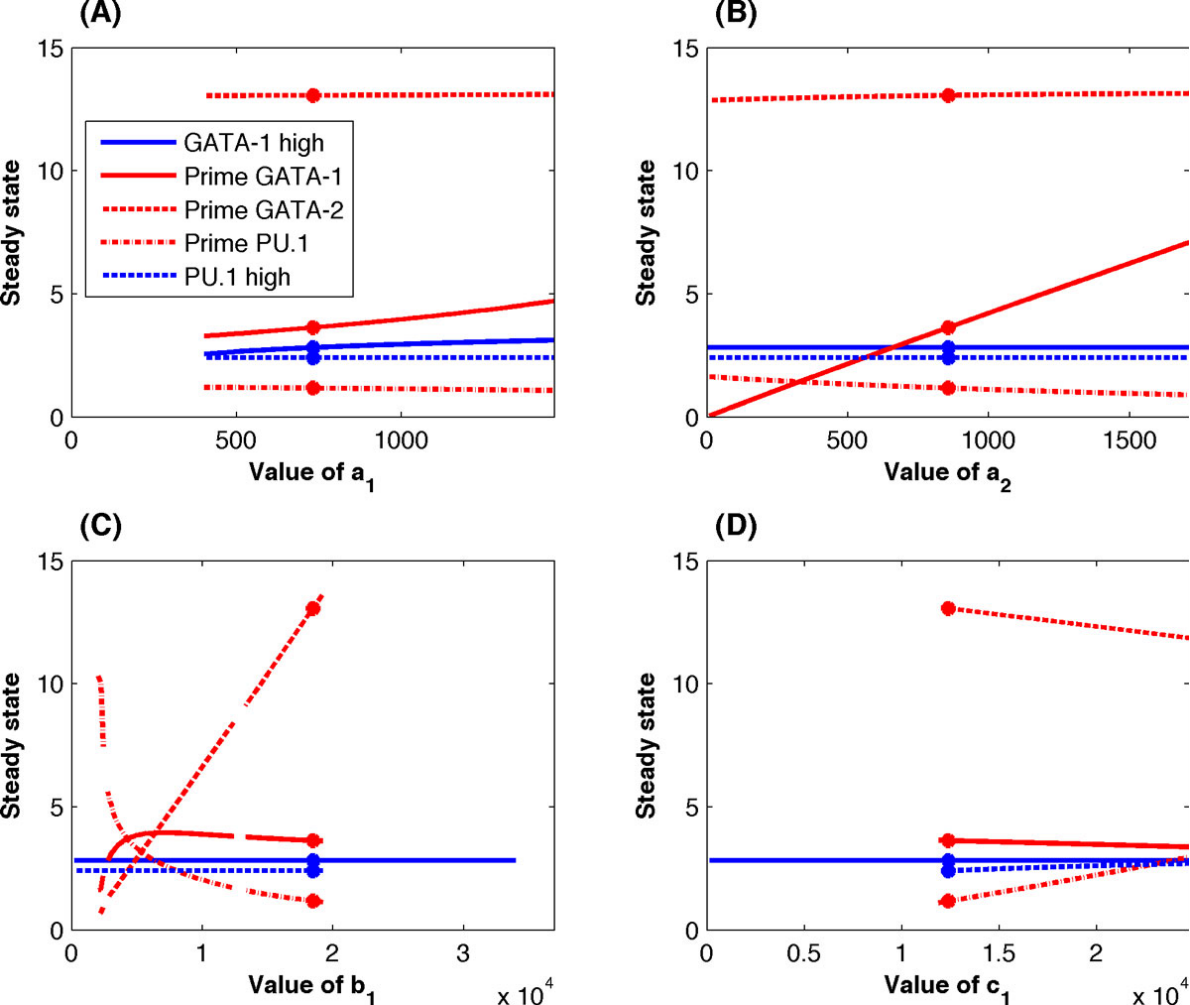
**Bifurcation analysis of the four synthesis rates**. Tristability property of the system when one of the synthesis rates varies. (A) Rate constant *a*_1_; (B) rate constant *a*_2_; (C) rate constant *b*_1_; and (D) rate constant *c*_1_. The curves show the value of a model parameter with which the system maintains three stable steady states. (Blue solid line: the steady state with high GATA-1 level; blue dash line: the steady state with high PU.1 level; red solid/dash/dash-dot line is the level of GATA-1/GATA-2/PU.1 in the primed state).

An important question in the estimation of model parameters is to what extent the model with the varied parameter still maintains the three stable steady states. To answer this question, we examined the tristability of the proposed model (Eq. 23) under the variations of one model parameter. For each rate constant, the perturbed values range from 0 to the 2-fold of the estimated value. We first tested the influence of the four synthesis rates *a*_1_, *a*_2_, *b*_1 _and *c*_1 _since these parameters are important in regulating the expression of these three genes. Figure 5 shows that the tristability property is not sensitive to the change of synthesis rate *a*_2_, which suggested that the expression of GATA-1 is not sensitive to the regulation of GATA-2. The result is consistent with the experimental observation showing that this regulation is relatively weaker than other genetic regulations in the system. Interestingly, the system maintains tristability if the synthesis rate of GATA-1 (*a*_1_) or PU.1 (*c*_1_) increased, or the synthesis rate of GATA-2 (*b*_1_) decreased. However, the tristability property of the system disappears when these three rate constants vary along the opposite direction, in particular for the synthesis rates of GATA-2 and PU.1. The discontinuity of the curves in Figure 5C suggested that the tristability property of the system disappears when the value of *b*_1 _is below a threshold value.

### Stochastic dynamics

We have successfully realized the three steady states of the HSCs and genetic switching using the genetic regulations between GATA genes and gene *PU.1*. However, the proposed deterministic model failed to describe the heterogeneity in the differentiation decision of the HSCs. To tackle the challenge, we designed a stochastic model to realize the function of intrinsic noise arising from key species with small copy numbers, such as DNA and mRNA. Using our proposed stochastic modelling approach [50], we designed the following stochastic model based on the developed deterministic model (Eq. 1), given by

(9) $$\begin{array}{c} X\left(t+\tau \right)=X\left(t\right)+P\left[\frac{a_{1}X+a_{2}Y}{a_{3}+a_{4}X+a_{5}Y+a_{6}Z+a_{7}XZ}\tau \right]-P\left[k_{1}X\tau \right]+P\left[\mu k_{2}^{*}Y\tau \right]\\ Y\left(t+\tau \right)=Y\left(t\right)+P\left[\frac{b_{1}Y}{b_{2}+b_{3}X+b_{4}Y+b_{5}Z+b_{6}YZ}\tau \right]-P\left[k_{2}Y\tau \right]-P\left[k_{2}^{*}Y\tau \right]\\ Z\left(t+\tau \right)=Z\left(t\right)+P\left[\frac{c_{1}Z}{c_{2}+c_{3}X+c_{4}Y+c_{5}Z+c_{6}XZ+c_{7}YZ}\tau \right]-P\left[k_{3}Z\tau \right] \end{array}$$

where *X*, *Y*, and *Z *are the copy numbers of protein GATA-1, GATA-2 and PU.1 respectively,  τ is the stepsize, *P*(*λ*) is a Poisson random variable with mean *λ*. Protein concentrations in model Eq. (1) were transferred into the molecular copy numbers in this stochastic model. To reduce the computing time of stochastic simulations, the mechanisms of genetic switching were realized in the time period

(10) $$k_{2}^{*}=\left\{\begin{array}{c} 25\;\;\;\;50\leq t\leq 200\\ \;0\;\;\;\;\;\;\;\;\;\;\;\text{else} \end{array}\right).$$

**Figure 6:**
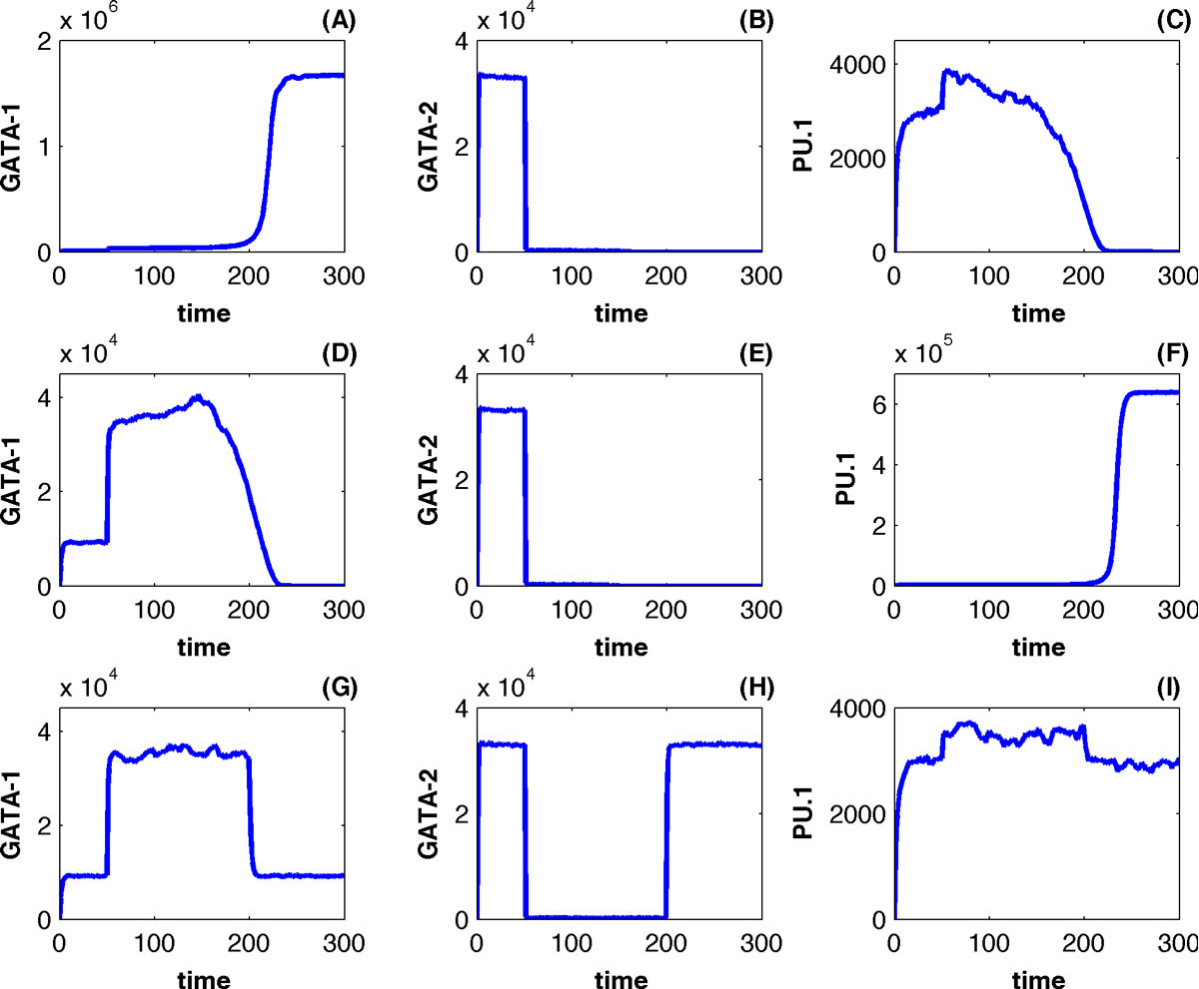
**Genetic switching of the stochastic model using the same model parameters**. The switching mechanism was realized by using $k_{2}^{*}$ = 25, *μ *= 0.28 during the time period [50, 200]. (A, B, C) A successful switching leading to the erythrocyte lineage; (D, E, F) a successful switching leading to the granulocyte lineage; (G, H, I) an unsuccessful switching maintained at the primed state.

Figure 6 gives three stochastic simulations using the same rate constants in Figure 4 and *μ *= 0.28. At time point *t *= 50, the expression level of GATA-2 decreased quickly to the basal level due to the large degradation rate $k_{2}^{*}$. Although the expression levels of genes *GATA-1 *and *PU.1 *increased quickly from time *t *= 50, the high expression level of one gene inhibited the expression of the other gene. Figures 6A, 6B and 6C show that the high expression levels of *GATA-1 *inhibited the expression of gene *PU.1 *and *GATA-2*, leading the cell into the erythrocyte lineage; whereas the expression levels of *GATA-1 *failed to increase further in Figures 6D and its expression was inhibited by the growing expression levels of *PU.1 *in Figure 6F, leading the cell into the granulocyte lineage. In addition, it was possible that the expression levels of neither gene *GATA-1 *nor *PU.1 *were high enough to inhibit the expression of the other gene. Figures 6G, 6H and 6I gave a simulation of unsuccessful switching in which the expression levels of these three genes maintained at an intermediate state that was determined by the value of *μ*. When the degradation rate of GATA-2 returned to the normal value at *t *= 300, the expression levels of these three genes returned to the original priming state.

**Figure 7:**
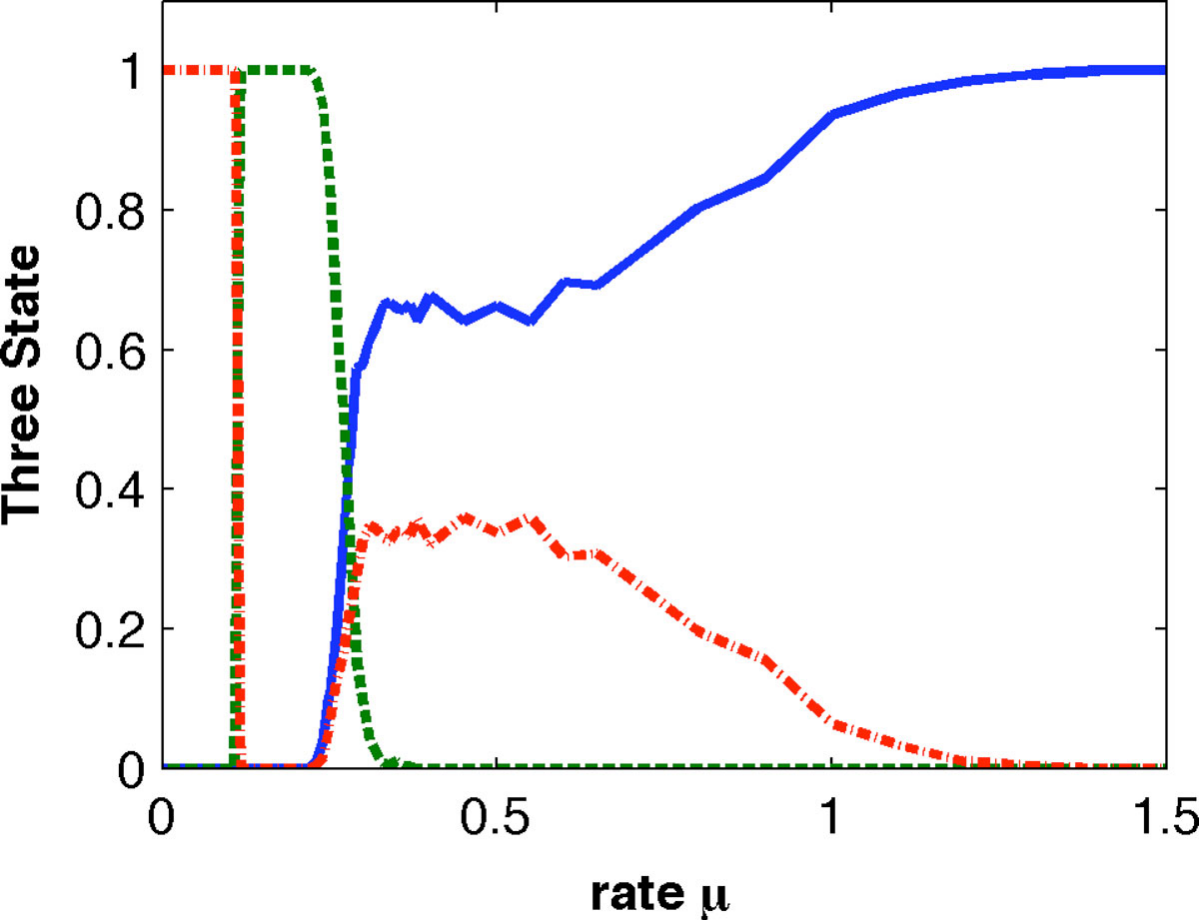
**Fractions of cells showing different lineage states based on different values of the rate constant *μ *and 1000 simulations**. (blue solid line: system showing the erythrocyte lineage; green dash line: system showing the priming state; red dash-dot line: system showing the granulocyte lineage).

To obtain the statistical property of genetic switching, we generated 1000 stochastic simulations for each value of *μ *over the range of [0, 1.5]. When the value is small (*μ *< 0.11), which mimicked the experimental condition of gene *GATA-2 *knock-down, Figure 7 shows that all simulations have high expression levels of gene *PU.1*. On the contrary, when the value of *μ *is large (*μ *≥ 1.4), all simulations were switched to the state with high expression levels of gene *GATA-1*, which represents the mechanisms of GATA switch that lead HSCs to the erythrocyte lineage. In addition, there is a range of values (0.12 <*μ *≤ 0.22) with which the system failed to realize genetic switching and all stochastic simulations stay at the priming state. When the value of *μ *locates between these windows, stochastic simulations can realize two or three different steady states. For example, when the value of *μ *is 0.11 ≤ *μ *≤ 0.12, the system state is in either the priming state or granulocyte lineage. An important prediction in Figure 7 is that, when the value of *μ *is (0.22 <*μ *≤ 0.37), stochastic simulation of the system may lead to anyone of the three steady states, namely the erythrocyte, priming and granulocyte lineages, such as the case demonstrated in Figure 6.

## Discussion

In this work we proposed a mathematical model to study the mechanisms of the GATA-PU.1 gene network in the determination of HSC differentiation pathways. The novelty of this model is the inclusion of gene *GATA-2 *and the GATA switch model based on the experimentally determined regulatory mechanisms. Our simulation results suggested that, based on the experimental determined regulatory mechanisms, the addition of the third gene, namely gene *GATA-2*, is necessary and adequate to realize the three stable steady states of the HSCs. This result is consistent with the prediction that a connector gene *X *is required to realize the primed state [15]. Although the third gene negatively regulates the expression of *PU.1 *in these two models, the regulatory mechanisms of the third gene in these two models are not completely the same. For example, it was assumed that gene *GATA-1 *positively regulate the expression of the unknown gene *X *[15], rather than the negative regulation of gene *GATA-2 *by gene *GATA-1 *in this work. Compared with the model in [15] in which additional and unknown external signals are required to maintain the tristability property, regulations between these three genes in our proposed model are adequate to maintain the tristability property of the system, which represents a successful approach in utilizing experimentally confirmed regulatory mechanisms to realize tristability property of the HSCs in regulating the erythroid-myeloid lineage decision.

The proposed model in this work for a network of three genes is a general framework that includes a recently published model for a network of two genes as a special case [18]. Unlike the model of two genes, which realized tristability using the bifurcation of the system by increasing the ratio of two model parameters, our model maintains the tristability property over a wide range of model parameter values. A related interesting question is the minimal motif to realize the stability property of a regulatory network with different numbers of steady states. It has been demonstrated that the two-gene module with self-activation and mutual repression can realize stability property with two steady states [50-53]. Although attempts have been made to realize tristability property of this two-gene module using various assumptions of regulatory mechanisms [13,14,17], our research demonstrated that a regulatory module with three genes could maintain stability property with three steady states without the assumption of the autoregulation via high order multimers. A similar result is that a three-component motif with four links can realize tristability [54]. However, the GATA-PU.1 module has six links together with more complex regulatory mechanisms. It would be interesting to analyze theoretically the stability property of a regulatory module with the maximal number of steady states under various regulatory mechanisms.

Stochastic simulations in this work predicted that the synthesis rate of GATA-1 during the decreasing process of GATA-2 determines the probability of erythroid-myeloid lineage decision. Since this synthesis rate represents the availability of GATA-1 in the genomic regulatory regions during the GATA switch, there are two potential resources of noise to explain the variations of GATA-1 proteins in the genomic regions. First, recent experimental research investigating cellular processes at single cells has revealed convincing evidence showing large heterogeneity in protein abundance and dynamics among genetically identical cells [55]. The variations of protein concentration in different cells may lead to different rates for GATA-1 to enter the regulatory regions during the process of GATA switch. The heterogeneity in protein abundance may be one of the reasons to explain the differentiation preference of HSCs for the erythroid lineage or the myeloid lineage. In addition, the intrinsic noise due to the small copy numbers of molecular species in the GATA-PU.1 module may further contribute the heterogeneity to the HSC lineage differentiation decision even for cells having the same lineage decision preference. These various resources of noise in gene expression may be part of the transcriptome-wide noise proposed by Huang and colleagues [32]. Therefore more comprehensive stochastic models are needed to explain the functions of other types of noise in the decision of lineage selection.

## Conclusions

In summary, we proposed a mathematical model to study the mechanisms of the GATA-PU.1 gene network in the determination of HSC differentiation pathways. In addition, a multiple-objective optimization approach was developed to infer model parameters in order to realize the three stable steady states representing the three different types of blood cells and genetic switching. A stochastic model was also designed to describe the function of noise in determining the differentiation pathways. Stochastic simulations successfully realized different proportions of cells leading to different developmental pathways under the same experimental conditions, and provided testable predictions regarding the conditions and mechanisms to realize different differentiation pathways. This work represents the first attempt at using a discrete stochastic model to realize the decision of HSC differentiation pathways with a multimodal distribution.

## Competing interests

The authors declare that they have no competing interests.

## Authors' contributions

T.T conceived the research. T.T. and K.S-M. conducted research, analyzed data and wrote the manuscript. All authors read and approved the final manuscript.

## Supplementary Material

Additional file 1 **Supplementary Information**. It provides the detailed assumptions of the mathematical model, the list of chemical reactions, and proof of Theorem 1 in the paper. Click here for file

## Declarations

The publication costs for this article were funded by the corresponding author.

This article has been published as part of *BMC Systems Biology *Volume 8 Supplement 1, 2014: Selected articles from the Twelfth Asia Pacific Bioinformatics Conference (APBC 2014): Systems Biology. The full contents of the supplement are available online at http://www.biomedcentral.com/bmcsystbiol/supplements/8/S1.
