# Supplementary material for: Mathematical modeling of GATA-switching for regulating the differentiation of hematopoietic stem cell
Source: BMC Syst Biol. 2014 Jan 24;8(Suppl 1):S8. doi: 10.1186/1752-0509-8-S1-S8 (PMC4080254; doi:10.1186/1752-0509-8-S1-S8)
Supplement: Additional file 1 — Supplementary Information. It provides the detailed assumptions of the mathematical model, the list of chemical reactions, and proof of Theorem 1 in the paper. [file 1752-0509-8-S1-S8-S1.pdf]

Supporting Material

Mathematical modelling of GATA switch in regulating hematopoietic stem cell differentiation

Tianhai Tian and Kate Smith-Miles

This supporting material first gives modelling assumptions and chemical reactions that are listed in Section 1. Section 2 proves the stability conditions of the three steady states of the mathematical model.

Section 1. Assumptions of the mathematical model

It was proposed that each of the three genes *GATA-1, GATA-2* and *PU.1* forms a positive auto-regulation loop and promote its expression,

$$x + D_x \underset{d1}{\overset{a1}{\rightleftarrows}} x\text{-}D_x \tag{1}$$

$$y + D_y \underset{d2}{\overset{a2}{\rightleftarrows}} y\text{-}D_y \tag{2}$$

$$z + D_z \underset{d3}{\overset{a3}{\rightleftarrows}} z\text{-}D_z \tag{3}$$

where x, y and z represent GATA-1, GATA-2 and PU.1 proteins, respectively, D_x, D_y and D_z are the corresponding DNA of each gene. Here $x + D_x$ represents the binding reaction of protein x to its DNA promoter site D_x, while $x\text{-}D_x$ denotes the molecular complex of protein x and its DNA promoter site D_x. In addition, GATA-2 can bind to the GATA-1 DNA promoter site to weakly promote the expression of GATA-1 in order to maintain a moderate level of GATA-1 protein in the primed state,

$$y + D_x \underset{d4}{\overset{a4}{\rightleftarrows}} y\text{-}D_x \tag{4}$$

However, there is mutual negative regulation between the GATA genes and PU.1 gene. It has been showed that GATA-1 inhibits gene *PU.1* expression via not only protein-protein interaction to prevent the binding of PU.1 to its cofactor c-Jun [1] but also physical binding of GATA-1 to the *PU.1* gene at the promoter region [2]. It was assumed that GATA-2 has similar regulatory mechanisms as GATA-1 to inhibit the expression of gene *PU.1*. In addition, PU.1 represses GATA-1 by binding to it on DNA [3] and also forming the GATA-1-PU.1 complex to inhibit GATA-1 binding to its DNA [4]. Following the assumptions in [5], it was assumed that PU.1 inhibits the expression of gene GATA-1 and GATA-2 by the binding of GATA-PU.1

heterodimers to GATA DNA promoters. These regulatory mechanisms are represented by the formation of heterodimers

$$x+z \underset{d5}{\overset{a5}{\rightleftarrows}} xz, \tag{5}$$

$$y+z \underset{d6}{\overset{a6}{\rightleftarrows}} yz, \tag{6}$$

and protein-DNA binding reactions

$$x+D_y \underset{d7}{\overset{a7}{\rightleftarrows}} x\text{-}D_y \tag{7}$$

$$x+D_z \underset{d8}{\overset{a8}{\rightleftarrows}} x\text{-}D_z \tag{8}$$

$$y+D_z \underset{d9}{\overset{a9}{\rightleftarrows}} y\text{-}D_z \tag{9}$$

$$z+D_x \underset{d10}{\overset{a10}{\rightleftarrows}} z\text{-}D_x \tag{10}$$

$$z+D_y \underset{d11}{\overset{a11}{\rightleftarrows}} z\text{-}D_y \tag{11}$$

as well as binding reactions of hetero-dimers to the DNA promoter regions

$$xz+D_x \underset{d12}{\overset{a12}{\rightleftarrows}} xz\text{-}D_x \tag{12}$$

$$yz+D_y \underset{d13}{\overset{a13}{\rightleftarrows}} yz\text{-}D_y \tag{13}$$

$$xz+D_z \underset{d14}{\overset{a14}{\rightleftarrows}} xz\text{-}D_z \tag{14}$$

$$yz+D_z \underset{d15}{\overset{a15}{\rightleftarrows}} yz\text{-}D_z \tag{15}$$

In this work the detailed transcription process and mRNA molecules were excluded from the model. We used a simplified process to represent protein synthesis,

$$x\text{-}D_x \xrightarrow{k_1} x+x\text{-}D_x \tag{16}$$

$$y\text{-}D_y \xrightarrow{k_2} y+y\text{-}D_y \tag{17}$$

$$z\text{-}D_z \xrightarrow{k_3} z+z\text{-}D_z \tag{18}$$

$$y\text{-}D_x \xrightarrow{k_4} x+y\text{-}D_x \tag{19}$$

In addition, protein degradation was represented by the first order reactions

$$x \xrightarrow{k_5} () \tag{20}$$

$$y \xrightarrow{k_6} () \tag{21}$$

$$z \xrightarrow{k_7} (). \tag{22}$$

Biochemical reactions in this system are classified into fast reactions that are assumed to be in an equilibrium state and slow reactions that represent transcription and degradation. Fast reactions include heterodimer formation and DNA binding reactions (Eq. 1~25). For example, reactions (Eq. 11) and (Eq. 22) can be simplified as

$$[\text{x-D}_\text{x}] = \mu_1 [\text{x}][\text{D}_\text{x}]$$

$$[\text{xz-D}_\text{x}] = = \mu_2 [\text{xz}][\text{D}_\text{x}] = = \mu_3 [\text{x}][\text{z}][\text{D}_\text{x}].$$

Since the total concentration of the protein remains constant, namely

$$\begin{aligned}[D_0] &= [\text{D}_\text{x}]+[\text{x-D}_\text{x}]+[\text{y-D}_\text{x}]+[\text{z-D}_\text{x}]+[\text{xz-D}_\text{x}] \\ &= (1 + a_4 x + a_5 y + a_6 z + a_7 xz)[\text{D}_\text{x}],\end{aligned}$$

we develop a mathematical model (Eq. 23) to describe the expression dynamics of genes GATA-1, GATA-2 and PU.1, given by

$$\begin{aligned}\frac{dx}{dt} &= \frac{a_1 x + a_2 y}{a_3 + a_4 x + a_5 y + a_6 z + a_7 xz} - k_1 x \\ \frac{dy}{dt} &= \frac{b_1 y}{b_2 + b_3 x + b_4 y + b_5 z + b_6 yz} - k_2 y \\ \frac{dc}{dt} &= \frac{c_1 z}{c_2 + c_3 x + c_4 y + c_5 z + c_6 xz + c_7 yz} - k_3 z\end{aligned} \tag{23}$$

Section 2. Stability conditions of the steady states

The steady states of the proposed model (Eq. 23) are given by the solutions of the nonlinear equation

$$\begin{aligned}F_1(x,y,z) &= \frac{a_1 x + a_2 y}{a_3 + a_4 x + a_5 y + a_6 z + a_7 xz} - k_1 x \\ F_2(x,y,z) &= \frac{b_1 y}{b_2 + b_3 x + b_4 y + b_5 z + b_6 yz} - k_2 y \\ F_3(x,y,z) &= \frac{c_1 z}{c_2 + c_3 x + c_4 y + c_5 z + c_6 xz + c_7 yz} - k_3 z\end{aligned} \tag{24}$$

This system may have up to four steady states. One can verify that the system has the following three steady states

$$(x_0, y_0, z_0) = (0,0,0) \tag{25}$$

$$(x_1, y_1, z_1) = (\frac{a_1 - k_1 a_3}{k_1 a_4}, 0, 0) \tag{26}$$

$$(x_2, y_2, z_2) = (0, 0, \frac{c_1 - k_3 c_2}{k_3 c_5}) \tag{27}$$

We can verify the following conditions for the existence of stable steady state.

Theorem 1. (1) The trivial steady state (Eq. 25) is unstable if any one of the following conditions is satisfied

$$\frac{a_1}{a_3} > k_1,\ \frac{b_1}{b_2} > k_2,\ \frac{c_1}{c_2} > k_3 \tag{28}$$

(2) The steady state with high expression level of gene GATA-1 (Eq. 26) is stable if the following conditions are satisfied

$$\frac{b_1}{(b_2 + b_3 x_1)} < k_2,\quad \frac{c_1}{(c_2 + c_3 x_1)} < k_3 \tag{29}$$

(3) The steady state with a high expression level of gene PU.1 (Eq. 27) is stable if the following conditions are satisfied

$$\frac{a_1}{(a_3 + a_6 z_2)} < k_1,\quad \frac{b_1}{(b_2 + b_5 z_2)} < k_2 \tag{30}$$

Proof. (1) When $(x_0, y_0, z_0) = (0,0,0)$, the Jacobian matrix of the nonlinear system is

$$J_0 = \begin{bmatrix} \frac{a_1}{a_3} - k_1 & \frac{a_2}{a_3} & 0 \\ 0 & \frac{b_1}{b_2} - k_2 & 0 \\ 0 & 0 & \frac{c_1}{c_2} - k_3 \end{bmatrix}.$$

When any one of the conditions in (Eq. 28) is satisfied, at least one of the eigenvalues of the Jacobian matrix is positive.

(2) Under the conditions y=0 and z=0, the second and third equations in (Eq. 24) are equal to zero and the first equation becomes

$$\frac{a_1 x}{a_3 + a_4 x} = k_1 x$$

By assuming $x \neq 0$, then state (Eq. 26) is a steady state. The Jacobian matrix of the nonlinear system (Eq. 24) for this steady state is

$$J_1 = \begin{bmatrix} \frac{a_1(a_3+a_4x_1)-a_1a_4x_1}{(a_3+a_4x_1)^2}-k_1 & \frac{a_1(a_3+a_4x_1)-a_1a_5x_1}{(a_3+a_4x_1)^2} & \frac{-a_1x_1(a_6+a_7x_1)}{(a_3+a_4x_1)^2} \\ 0 & \frac{b_1}{(b_2+b_3x_1)}-k_2 & 0 \\ 0 & 0 & \frac{c_1}{(c_2+c_3x_1)}-k_3 \end{bmatrix}$$

The three eigenvalues of the Jacobian matrix are

$$\lambda_1 = \frac{-a_1a_4x_1}{(a_3+a_4x_1)^2}, \quad \lambda_2 = \frac{b_1}{(b_2+b_3x_1)}-k_2, \quad \lambda_3 = \frac{c_1}{(c_2+c_3x_1)}-k_3,$$

It is clear that $\lambda_1 < 0$. When conditions (Eq. 29) are satisfied, we have $\lambda_2 < 0$ and $\lambda_3 < 0$. Therefore this steady state is stable.

(3) Under the conditions x=0 and y=0, the first and second equations in the nonlinear system (Eq. 24) are equal to zero and the third equation is

$$\frac{c_1z}{c_2+c_5z} = k_3z$$

By assuming $z \neq 0$, then state (Eq. 27) is a steady state. The Jacobian matrix of the nonlinear system (Eq. 24) for this steady state is

$$J_1 = \begin{bmatrix} \frac{a_1}{a_3+a_6z_2}-k_1 & \frac{a_2}{a_3+a_6z_2} & 0 \\ 0 & \frac{b_1}{(b_2+b_5z_2)}-k_2 & 0 \\ \frac{-c_1z_2(c_3+c_6z_2)}{(c_2+c_5z_2)^2} & \frac{-c_1z_2(c_4+c_7z_2)}{(c_2+c_5z_2)^2} & \frac{c_1(c_2+c_5z_2)-c_1c_5z_2}{(c_2+c_5z_2)^2}-k_3 \end{bmatrix}$$

The three eigenvalues of the Jacobian matrix are

$$\lambda_1 = \frac{a_1}{a_3+a_6z_2}-k_1, \quad \lambda_2 = \frac{b_1}{(b_2+b_5z_2)}-k_2, \quad \lambda_3 = \frac{c_1(c_2+c_5z_2)-c_1c_5z_2}{(c_2+c_5z_2)^2}-k_3.$$

It is clear that $\lambda_3 < 0$ when condition (Eq. 30) is satisfied. Under the condition (Eq. 30), we can conclude that $\lambda_1 < 0$ and $\lambda_2 < 0$. Therefore this steady state is stable.

References

1. Ferreira R, Ohneda K, Yamamoto M, Philipsen S: **GATA1 function, a paradigm for transcription factors in hematopoiesis**. *Mol Cell Biol* 2005, **25**(4):1215-1227.
2. Chou ST, Khandros E, Bailey LC, Nichols KE, Vakoc CR, Yao Y, Huang Z, Crispino JD, Hardison RC, Blobel GA *et al*: **Graded repression of PU.1/Sfpi1 gene transcription by GATA factors regulates hematopoietic cell fate**. *Blood* 2009, **114**(5):983-994.
3. Stopka T, Amanatullah DF, Papetti M, Skoultchi AI: **PU.1 inhibits the erythroid program by binding to GATA-1 on DNA and creating a repressive chromatin structure**. *Embo J* 2005, **24**(21):3712-3723.
4. Zhang P, Zhang X, Iwama A, Yu C, Smith KA, Mueller BU, Narravula S, Torbett BE, Orkin SH, Tenen DG: **PU.1 inhibits GATA-1 function and erythroid differentiation by blocking GATA-1 DNA binding**. *Blood* 2000, **96**(8):2641-2648.
5. Andrecut M, Halley JD, Winkler DA, Huang S: **A general model for binary cell fate decision gene circuits with degeneracy: indeterminacy and switch behavior in the absence of cooperativity**. *PLoS One* 2011, **6**(5):e19358.
